# Digestive tolerance and postprandial glycaemic and insulinaemic responses after consumption of dairy desserts containing maltitol and fructo-oligosaccharides in adults

**DOI:** 10.1038/ejcn.2014.30

**Published:** 2014-03-19

**Authors:** F Respondek, C Hilpipre, P Chauveau, M Cazaubiel, D Gendre, C Maudet, A Wagner

**Affiliations:** 1 Innovation Department, Tereos Syral, Marckolsheim, France; 2 Biofortis SAS, Saint-Herblain, France

## Abstract

**Background/objectives:**

To evaluate the short-term digestive tolerance and glycaemic response of several associations of maltitol and short-chain fructo-oligosaccharides (scFOS) used to replace sugars (for example, dextrose) in foods.

**Subjects/methods:**

Thirty-six healthy subjects aged 18–60 years were recruited for the study and 32 completed it. The subjects consumed six different mixtures of dextrose, maltitol and scFOS added in a chocolate dairy dessert at a dosage of 35 g. The test days were separated by 2-week washout periods. The subjects reported the intensity of four individual gastrointestinal (GI) symptoms, number of bowel movements and stool frequency for the 48 h following consumption of the dessert. A subgroup of 18 subjects also provided blood samples 2 h after intake to evaluate the postprandial glycaemic and insulinaemic responses.

**Results:**

The composite score calculated from the intensity of flatulence, borborygmi, bloating and discomfort was significantly higher (*P*<0.0001) for all the desserts containing maltitol and/or scFOS than for the control dessert containing dextrose, but remains at the level of mild effects. The number of bowel movements was also slightly increased (*P*=0.0006) and the stools were softer (*P*=0.0045) for the first 24 h but not after (*P*=0.1373 and 0.5420, respectively). Blood glycaemic and insulinaemic responses were lower for all the sugar-free recipes containing maltitol and scFOS in comparison to the control one (*P*<0.0001).

**Conclusions:**

This study has shown that maltitol and scFOS can be used jointly when formulating sugar-free foods with the benefit to lower postprandial glycaemic response with only a small and transient increase in non-serious GI symptoms.

## Introduction

Maltitol belongs to polyol family that are sugar-free sweeteners also called ‘sugar alcohols', widely used in various foods either as sweetening agents in sugar-free foods or energy-reduced foods or for other technological purposes such as bulking agent.^1^ In the European regulation, that is, Council Directive on nutrition labelling for foodstuffs (90/496/EEC consolidated version 11.12.2008), ‘sugars' means all monosaccharides and disaccharides present in food but excludes polyols. Maltitol, as other polyols, has the advantage of a lower caloric intake than sugars (2.4 kcal vs 4 kcal per g) and a reduced glycaemic response.^2^ Other types of food ingredients such as dietary fibres are also used to reduce sugar content or energy in foods. These fibres are not digested in the small intestine and will be intact when reaching the colon where some of them will be fermented. In Europe, all fibres are considered to provide 2 kcal of energy per g. For example, short-chain fructo-oligosaccharides (scFOS) produced from sucrose are prebiotic fibres that will be selectively fermented by the intestinal microbiota, providing potential health benefits to the host.^3^ Although being not hydrolysed by the digestive enzymes present in the small intestine and not absorbed or only partially absorbed, these low-digestible carbohydrates (LDCs) are useful to reduce postprandial blood glucose response that is a beneficial physiological effect.^4,5^ As a result, LDCs may also affect laxation and cause non-serious and transient gastrointestinal (GI) effects, especially with high or excessive intakes, sometimes limiting the acceptance of food products containing them despite their health benefits. Digestive symptoms linked to the consumption of little or no digestible carbohydrates are generally assessed product by product and are rather well known for maltitol^6,7^ and scFOS.^8,9^ However, several LDCs are often mixed in foods and very limited scientific literature documents the effect of these LDC mixtures on GI symptoms.^10^

The primary objective of the present study was to check at once the short-term digestive tolerance of several associations of maltitol and scFOS, allowing a complete replacement of sugar (for example, dextrose) content of a food matrix, in healthy adults. The secondary objective was to assess the effect of these associations on glycaemic and insulinaemic responses in comparison with a control formulation.

## Subjects and methods

### Subjects

Thirty-six healthy subjects were recruited for this study according to the following inclusion criteria: age (18–60 years), body mass index (18.5–30.0 kg/m^2^), stool frequency within normal range (between three bowel movements a week to three bowel movements a day), not suffering from a metabolic, functional or inflammatory disease affecting intestinal transit and nutrient absorption, not suffering from irritable bowel syndrome according to ROME III criteria, not using medication that could affect nutrient absorption, lipid or carbohydrate metabolism. Among them, 18 subjects also agreed to take part in the assessment of glycaemic and insulinaemic responses. The subjects were screened according to fasting blood glucose level ⩽1.1 g/l, normal renal function and no clinically significant abnormality concerning complete blood count, and liver enzymes.

All the subjects provided a written informed consent to participate after the study procedures had been explained to them. The study was approved by the ethics committee (CPP OuestIV, Nantes, France) and was performed in accordance with the guidelines of the International Conference on Harmonisation of Good Clinical Practice and the principles laid down in the current version of the Declaration of Helsinki.

### Experimental design

This randomised, double-blind reference-controlled, six-period cross-over study was performed in a single clinical site (Biofortis, Nantes, France). The six different dairy desserts were randomly administered orally to the subjects during six different experimental sessions with at least 2 washout weeks between them. A randomisation table was generated using SAS software version 9.3 (SAS Institute Inc., Cary, NC, USA). A minimum of 2 weeks was kept between each test. During each experimental visit (V1–V6), the subjects arrived at the clinical site after 12 h fasting and were subjected to a clinical examination and a medical enquiry. For the subjects participating in the tolerance test only, the test meal started directly after the clinical examination at T0. When relevant, the tolerance questionnaires filled since the last visit were recovered. For the monitoring at 24 and 48 h after ingestion, each subject received one diet questionnaire for 2 days, two Bristol stool scale questionnaires and two GI symptom questionnaires. In addition, the subjects were instructed not to eat foods favouring intestinal transit or rich in fermentable carbohydrates, prebiotics, polyols or aspartame, not to eat meals rich in fats and carbohydrates, not to drink alcoholic drinks and not to practise intense physical activity within 48 h before and after consuming each dairy dessert.

### Products being studied

The studied polyol was a maltitol (Maltilite P 200, Tereos Syral, Marckolsheim, France). The studied scFOS were FOS from sucrose (Actilight 950P, Beghin Meiji, Marckolsheim, France), comprising about 37% 1-kestose (GF2), 53% nystose (GF3) and 10% 1 F-β-fructofuranosyl nystose (GF4). Five mixtures at different doses and ratios of maltitol and scFOS were tested against a single dose of dextrose used as a reference. The studied products have been orally administered under a chocolate dessert cream form containing in decreasing order of weight importance: cocoa powder, maltodextrines, modified starch, milk proteins, chocolate flavour, carrageenans and sucralose for sweetness adjustment (Table 1).

### Evaluation of GI symptoms

During the screening phase, the subjects were asked to mention, when relevant, which symptoms they were having according to the ROME III criteria (including constipation, functional diarrhoea, so on.).^11^ The investigators recorded the possible consumption of foods containing polyols or fermentable carbohydrates 48 h before and 48 h after each study visit.

The evaluation of the digestive tolerance is based on the follow-up of four main GI symptoms, namely, flatulence, borborygm, bloating and abdominal pain/discomfort, and on the stool frequency and consistency after product consumption.^12^ The primary efficacy end point was the composite score of the four GI symptoms at 24 h. For each of these symptoms, the subject indicated their absence (score=0) or their presence over the last 24 h. If the symptom is present, the subject circled their intensity level on a Likert scale, graduated from 1 ‘very low' to 10 ‘very severe'. The sum of these four scores corresponds to the composite score of GI symptoms. Then, the scores of each individual GI score and composite score were classified in four arbitrary intensity categories to qualify digestive tolerance: intensity 0 for ‘absence', intensity 1 for ‘mild' (score ranging from 1 to 4), intensity 2 for ‘moderate' (scores ranging from 5 to 7) and intensity 3 for ‘severe' (score ranging from 8 to 10). The secondary end points were the number of bowel movements per 24 h and the stool consistency according to the validated Bristol stool scale^13^ within the 48 h following consumption of the products.

### Evaluation of the glycaemic and insulinaemic responses

For the subjects taking part in kinetics for glycaemia and insulinaemia, a nurse placed a catheter on the subject's arm, started the kinetic test for a 120-min period and then took off the catheter. The subjects were instructed to eat the dessert within 5–10 min in fasting conditions with 250 cl water at the clinical site. The kinetic test consists in sampling venous blood at times T5 and T1 min before the subject eats the meal, and then at T15, T30, T45, T60, T90, T120 min after the meal intake.

The blood samples were collected into sodium fluoride, potassium oxalate for glucose determination and EDTA tubes for insulin. The level of blood glucose was assessed by an enzymatic colorimetric method (Hitachi 911 and 912, Tokyo, Japan) and commercially available reagents (Boerhingher Mannheim GmbH Diagnostica, Mannheim, Germany). The blood insulin level was assessed by electrochemiluminescence (Elecsys, 2010, Roche Diagnostics, Meylan, France).

The subject's compliance was checked by the study coordinator during the sessions (meal intake according to the protocol and with respect to the sampling time).

### Statistical analyses

Summary results are presented as means±s.d. Variables were assessed for normality of distribution. If the normality assumption was violated, a log transformation of data was performed. A repeated measures analysis of variance model using visit, dessert type and visit × dessert type interaction as fixed effects was used to compare the effects of dairy desserts containing different formulations of maltitol and scFOS on the composite score of GI symptoms within the 24 h following consumption. In case of statistically significant product effect, a Dunnett's test was conducted to compare each test dessert with the control and a Tukey's test to compare each test dessert with all the desserts. The same statistical model was applied to evaluate the effects on secondary end points: composite score of GI symptoms at 48 h, individual GI symptoms at 24 and 48 h, stool frequency (number of bowel movements per 24 h) and stool consistency (according the Bristol stool scale). The incremental area under the curve (AUC) between 0 and 120 min (AUC_0–120_ _min_) for blood glucose and insulin concentrations was computed following the Food and Agriculture Organization recommendation.^14^ AUC_0–120_ _min_ and Cmax for glucose and insulin were analysed with an analysis of covariance (ANCOVA) model taking the baseline value for glucose or insulin accordingly as a covariate followed by a Dunnett's *post hoc* test (each test dessert was compared with a reference); in case of statistically significant product effect, a McNemar's test was performed to compare the frequency of adverse event between treatment groups.

A statistical analysis was conducted on both intention to treat (ITT) and per protocol (PP) populations using SAS software version 9.3 (SAS Institute Inc.). For all the statistical tests, the 0.05 level of significance was used, except for the interaction test with a 0.10 level, to claim a statistically significant effect.

## Results

### Study population

The ITT population represents the 36 randomised subjects and the PP population is composed of 32 randomised subjects who completed the study without any major deviation. Three subjects were excluded because they prematurely left the study without any specific reason and one subject was excluded because he took concomitant medications, which can have an impact on the results of the study. The ITT population had an average age of 41.3±12.8 years and an average body mass index of 23.25±2.74 kg/m^2^. They were 33% men and 66% women. The ITT population (*n*=18) who also participated in the evaluation of the glycaemic and insulinaemic responses had an average age of 39.0±12.6 years and an average body mass index of 23.38±2.87 kg/m^2^. Their average fasting glycaemia was 4.90±0.62 mmol/l and insulinaemia was 7.45±2.70 mU/l.

### Digestive tolerance questionnaires

The primary end point shows that GI symptom intensities are significantly higher with every mixture compared with the control dessert with 35 g dextrose in the ITT (*P*<0.0001, Table 2) and PP (*P*<0.0001, Supplementary Table 1) populations, but remains in a low range, as maximum average score is 10.8/40. Between 24 and 48 h, only desserts formulated with 35 g maltitol, 30 g maltitol and 5 g scFOS, and 17.5 g maltitol and 17.5 g scFOS still significantly induced more GI symptoms than the control one in the ITT population (*P*=0.0005; *P*=0.0353; *P*=0.0271, respectively). The symptoms are not statistically higher anymore in the PP population for this second period (Supplementary Table 1). No difference is seen between the different mixtures of maltitol and scFOS.

Individually analysing the symptoms constituting the composite score of GI symptoms (that is, flatulence, borborygmi, bloating, abdominal pain/discomfort) shows that only the complete replacement of dextrose by maltitol or by maltitol and scFOS and not its partial reduction (dessert with 24 g dextrose and 11 g scFOS) induces higher symptom scores for flatulence (*P*<0.0001), borborygmi (*P*<0.0001) and discomfort (*P*=0.0135) in the first 24 h following the intake of the dairy dessert in ITT population (Table 2). Only the dessert formulated with 17.5 g maltitol and 17.5 g scFOS induces a higher level of bloating in comparison with the control dessert (35 g dextrose) (*P*=0.0039). Between 24 and 48 h after the dessert intake, the flatulence score for the desserts with maltitol or maltitol with scFOS remained higher than the score for the control dessert, whereas most of the other symptom scores were not different anymore. Similar results are observed in PP population (Supplementary Table 1). During this period, only the dessert containing 35 g maltitol induced higher discomfort than the control dessert in the ITT (*P*=0.0295) but not in the PP populations.

### Stool frequency and consistency

Stool frequency is significantly increased within the 24 h following the intake of dairy desserts formulated with maltitol alone or maltitol with scFOS instead of dextrose in both ITT (*P*=0.0006) and PP (*P*=0.0016) populations. This difference with the control dessert is not seen anymore between 24 and 48 h (*P*=0.1373 and *P*=0.0947, respectively, for ITT and PP populations) (ITT: Table 3; PP: data not shown). A similar observation is made in the ITT population: the stools are slightly softer (*P*=0.0045) within the 24 h following intake of desserts but not afterwards (*P*=0.5420). No consistency difference is observed in the PP population within the 24 h (*P*=0.1084) or within the 48 h (*P*=0.9530) following the intake of the dairy desserts (Supplementary Table 2).

### Glycaemic and insulinaemic responses

**Figure 1:**
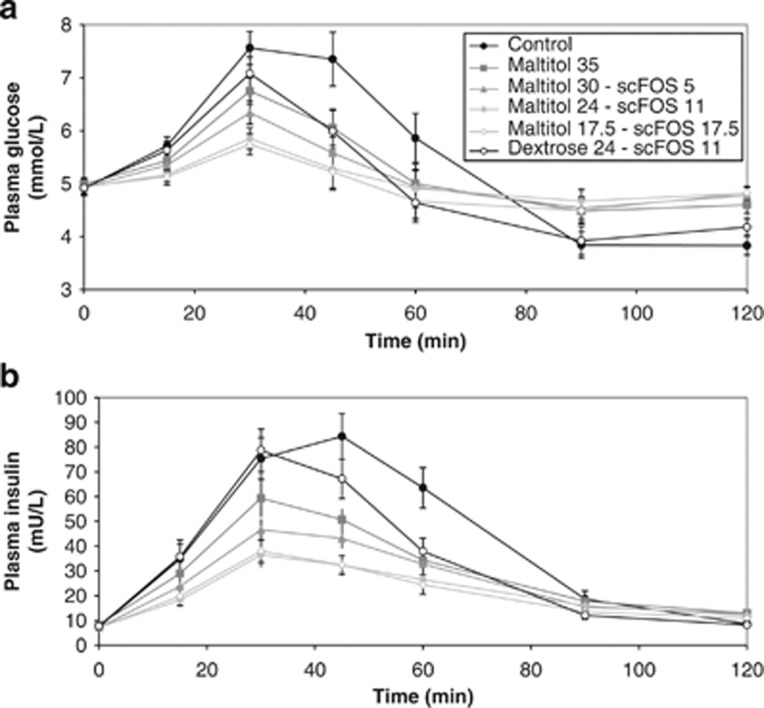
Plasma glucose (**a**) and plasma insulin (**b**) over 120 min after taking the dairy desserts containing 35 g dextrose or different mixtures of maltitol and scFOS in the ITT population (*n*=18). Data is expressed in mean±s.e.m.

All the dairy desserts formulated with maltitol and scFOS instead of dextrose induced a lower postprandial blood glucose response compared with dextrose in both ITT (Figure 1a) and PP (data not shown) populations as illustrated by lower AUC_0–120 min_ (*P*<0.0001) and lower peak (*P*<0.0001) of glycaemia (Table 4). In parallel, the insulin response is also lower for all the maltitol- and scFOS-formulated desserts compared with the control one (ITT population: Figure 1b and Table 4). The sugar-reduced dairy dessert containing 24 g of dextrose and 11 g of scFOS significantly lowers the peak of glycaemia as well as the insulin AUC_0–120 min_ but did not significantly reduce the glucose AUC_0–120 min_ (Dunnett's test: *P*=0.1113 for ITT, *n*=18 and *P*=0.0643 for PP, *n*=15) and had no effect on the peak of insulin (Dunnett's test: *P*=0.1611 for ITT and *P*=0.1121 for PP) (Table 4).

### Adverse events

Most of the adverse events reported during the study were not linked to the study product or to the research procedures. Only 11 adverse events out of 88 total events were linked to the research or to the products (imputability deemed possible). These adverse events were observed with the desserts containing the different mixtures of maltitol and scFOS and with the reference dessert, but no statistically significant association was observed between the ingredients and the imputability of adverse events. No serious adverse event was reported. Moreover, no diarrhoea was reported and digestive symptom intensity was mild.

## Discussion

Polyols are LDCs that are mainly used to replace sugars in foods such as confectionery, bakery and dairy products because they are non-cariogenic, have a low glycaemic response and lower caloric value than sugars.^2^ Dietary fibres such as scFOS are also used in foods to replace sugars^15^ because they have a sweet taste and also have a lower caloric value than digestible carbohydrates.^16,17^ Although these ingredients may have physiological benefits on dental health^18^ or on postprandial glycaemic response^2^ for maltitol and on digestive comfort^19^ for scFOS when consumed at an adequate level, they may induce transient and non-serious Gi symptoms when consumed in excessive amounts.^6,8^ Although digestive tolerance of each ingredient is well known, no data exist on the digestive tolerance of these ingredients jointly present in a food, whereas they may be complementary in term of rheological and sensorial properties.

This study aimed to evaluate in healthy adults the digestive tolerance and the postprandial glycaemic and insulinaemic responses of dairy desserts containing different mixtures of maltitol and scFOS in comparison with a control dessert containing 35 g dextrose. The dose of 35 g dextrose, to be replaced by 35 g maltitol in the sugar-free recipe, was chosen according to literature to induce significant GI symptoms after an occasional intake, but not to be at the laxative threshold estimated around 92 g.^6,10^ However, this intake level is much higher than the recent estimation of polyol intake per eating period when polyols are used for their sweetening properties as it averages 5.9 g and raises up to 11.8 g for the 95th percentile (Tennant D, Manuscript in preparation).

The sugar-reduced dairy dessert, formulated to contain 30% fewer dextrose than the reference dessert, only showed a higher GI score than the control one for the first 24 h after consumption. All the individual scores of symptoms as well as stool frequency and consistency were equivalent to the control for the 48 h following consumption. This might be explained by the fact that the scFOS intake was only 11 g, whereas excessive flatus usually appears above 30 g, borborygmi and bloating above 40 g and diarrhoea above 50 g scFOS per day.^8^ Contrary to the sugar-reduced dessert, all the other desserts with maltitol and scFOS induced more GI symptoms than the control one, but the symptoms were ‘mild' and transient in time as they were not significant anymore after 24 h.

The stool frequency was slightly higher and stools were a bit softer with all the desserts containing the different mixtures of maltitol alone and maltitol with scFOS than with the control one. However, the consistency was still within the range of ‘normal' consistency as it was scored between 3 and 4^13^ and average stool frequency remained below the diarrhoea threshold of three bowel movements per day.^11^ No difference was seen between the different mixtures of maltitol and scFOS in term of total GI score, individual symptoms, stool frequency or consistency. Few differences appear between some maltitol/scFOS desserts and the one reduced in dextrose with scFOS. Flatulence severity is more important with the two desserts containing 24 g maltitol/11 g scFOS and 17.5 g maltitol/17.5 g scFOS, inducing a slightly higher GI score than the sugar-reduced formula (24 g dextrose and 11 g scFOS) for the first 24 h. Flatulence is frequently the first symptom that appears when increasing the consumption of LDCs and it was verified for maltitol and scFOS.^10^

The stools obtained for the first 24 h were always slightly softer for all the formulations with maltitol/scFOS than for the one reduced in dextrose with scFOS. Although remaining in normal range, the stool frequency was also increased with the two desserts containing 35 g maltitol and 30 g maltitol+5 g scFOS in comparison with the dessert reduced in dextrose and containing 11 g scFOS. These differences may be explained by the higher presence of LDCs in these formulations, but also by the presence of maltitol that is known to increase the osmotic load in the intestine.^20^

In summary and accordingly to previous studies on other food types, the consumption of a sugar-free dairy dessert providing 35 g LDCs induces transient and mild GI symptoms. The severity and duration of symptoms were not really affected by the different tested mixtures of maltitol and scFOS in comparison with maltitol used alone. Most of the effects of maltitol on GI symptoms are linked to an increase in the osmotic pressure that some fibres may reduce by forming a gel of large molecules around sugar alcohols or by delaying gastric emptying.^21,22^ Some authors already cited a positive effect of FOS on the suppression of osmotic diarrhoea induced by maltitol but neither precise information nor possible mechanism was provided.^21^ As scFOS are soluble non-viscous fibres, they may not have an impact on the osmotic pressure induced by maltitol. However, as scFOS may have different metabolic fate than maltitol in the digestive tract, the digestive tolerances of each component do not interfere. In humans, scFOS stimulate the growth of bifidobacteria from 2.5–10 g/day and are completely fermented.^23^ At higher dosage, no further increase in faecal bifidobacteria is observed and flatulence is more frequent.^9^ These studies are however generally conducted over several days or weeks of consumption. A more recent study has shown that maltitol may also stimulate the growth of bifidobacteria and other bacterial groups but at a much higher level than scFOS, that is, around 45 g per day,^24^ which was not reached in our study.

### Glycaemic and insulinaemic responses

The postprandial glycaemic response of all the dairy desserts containing maltitol and/or scFOS is reduced in comparison with the glycaemic response of the control dairy dessert as illustrated by a lower AUC_0–120 min_ and/or lower peak of glycaemia. This lower glycaemic response was not due to hyperinsulinaemia induced by the ingredients used to replace dextrose. The postprandial insulin AUC_0–120 min_ and insulin peak are also reduced in comparison with the control dessert. This is in accordance with previous studies showing polyols in general, and therefore maltitol, have a low glycaemic and insulinaemic response allowing reducing postprandial glycaemic response when they replace sugars in foods and drinks.^2,25^ Indeed, only about 40% maltitol is absorbed in the small intestine and the leftover will be fermented in the colon.^2^ Similarly, in humans, scFOS are mostly not digested nor absorbed in the small intestine but completely fermented in the large intestine.^16^ In two subjects, it was previously shown that contrary to glucose, scFOS do not increase postprandial blood glucose or insulin.^26^ This study highlights that when used in place of sugars for partial or complete replacement, scFOS may contribute to lower the postprandial glycaemic and insulinaemic responses of foods as illustrated by a dairy dessert here. These effects could be of interest for the diets of people with impaired glucose tolerance or diabetes.^4^

In conclusion, this study confirms that reducing sugars and more particularly dextrose by adding polyols or fibres in foods has only a limited impact on GI symptoms in the short term. More importantly, it has shown that maltitol and scFOS can be used jointly when formulating sugar-free foods with the benefit to reduce postprandial glycaemic response with no further impact on GI symptoms than when maltitol is used alone.

## Figures and Tables

**Table 1:** Nutritional composition of the dairy desserts

*In g per 210* *g*	*Control*	*Dextrose 24 scFOS 11*	*Maltitol 35*	*Maltitol 30 scFOS 5*	*Maltitol 24 ScFOS 11*	*Maltitol 17.5 scFOS 17.5*
Calorie content (kcal)	218.9	193.0	162.9	159.3	154.8	151.6
Carbohydrates	48.1	32.1	48.1	41.6	32.1	25.9
Sugars^a^	36.0	25.3	1.1	1.2	1.2	1.6
Polyols	0.0	0.0	35.0	29.9	24	17.5
Lipids	2.2	3.0	2.2	2.5	3.0	3.0
Proteins	6.1	8.3	6.1	6.8	8.3	8.2
Fibres	2.7	14.8	2.7	8.0	14.8	21.3
scFOS	0.0	11.2	0.0	5.0	11.2	17.7

Abbreviation: scFOS, short-chain fructo-oligosaccharides.

a ‘Sugars' means all monosaccharides and disaccharides present in food but excludes polyols.

**Table 2:** Total and individual scores of gastrointestinal symptoms in the 24 and 48 h following consumption of dairy desserts containing 35 g dextrose (control dessert) or different mixtures of maltitol and scFOS in the ITT population (*n*=36)

		*Control*	*Dextrose 24 scFOS 11*	*Maltitol 35*	*Maltitol 30 scFOS 5*	*Maltitol 24 scFOS 11*	*Maltitol 17.5 scFOS 17.5*	*Dessert effect (*P*-value)*
0–24 h	Total score	3.15±4.26^a^	6.46±5.85^b^	8.86±6.49b,^c^	8.53±7.10b,^c^	9.41±6.99b,^c^	10.80±7.98b,^c^	<0.0001
	Flatulence	1.21±1.49	2.11±2.26	3.37±2.56*	2.74±2.50*	3.68±2.46*	4.11±2.34*	<0.0001
	Borborygmi	0.70±1.31	1.49±2.01	2.11±2.40*	2.41±2.43*	1.85±2.30*	2.43±2.40*	<0.0001
	Bloating	0.70±1.69	1.40±2.20	1.60±2.24	1.74±2.15	1.82±2.41	2.17±2.72*	0.0312
	Discomfort	0.55±1.68	1.46±2.41	1.77±2.44*	1.65±2.98	2.06±2.95*	2.09±2.70*	0.0135
								
24–48 h	Total score	0.88±1.71^a^	2.34±3.55a,^b^	3.60±5.39^b^	2.62±3.57^b^	2.24±3.37a,^b^	2.71±4.26^b^	0.0063
	Flatulence	0.27±0.80	0.83±1.50	1.37±1.99*	1.12±1.61*	1.06±1.54*	1.20±1.78*	0.0037
	Borborygmi	0.30±0.81	0.31±0.93	0.74±1.60	0.79±1.49	0.44±1.13	0.57±1.29	0.0823
	Bloating	0.12±0.55	0.49±1.17	0.69±1.64	0.35±0.92	0.47±1.02	0.57±1.36	0.2084
	Discomfort	0.18±0.58	0.71±1.54	0.80±1.64*	0.35±1.01	0.26±0.79	0.37±1.19	0.0448

Abbreviations: ITT, intention to treat; scFOS, short-chain fructo-oligosaccharides.

Each symptom is scored between 1 and 10; data is expressed in arbitrary unit mean±s.d.

a,b,c Desserts not sharing the same letter are significantly different (*P*<0.05, Dunnett's *post hoc* test vs the Control or Tukey's test among the five other mixtures).

* Significant increase (Dunnett's test) in gastrointestinal symptom scores compared with 35 g dextrose, *P*<0.05. Other observed differences (Tukey's test) for flatulence at 24 h: maltitol 24–scFOS 11 vs scFOS 11 (*P*=0.0197); maltitol 17.5–scFOS 17.5 vs scFOS 11 (*P*=0.0005); maltitol 17.5–scFOS 17.5 vs maltitol 30–scFOS 5 (*P*=0.0377).

**Table 3:** Stool frequency and consistency evaluated by the Bristol stool scale within the 24 and 48 h following consumption of the dairy desserts containing 35 g dextrose or different mixtures of maltitol and scFOS in the ITT population (*n*=36)

		*Control*	*Dextrose 24 scFOS 11*	*Maltitol 35*	*Maltitol 30 scFOS 5*	*Maltitol 24 scFOS 11*	*Maltitol 17.5 scFOS 17.5*	*Dessert effect (*P-*value)*
0–24 h	Frequency^a^	1.24±0.83	1.46±0.82	1.89±0.96^b^	1.91±0.93^b^	1.74±0.90^b^	1.74±0.89^b^	0.0006
	Consistency^c^	3.73±1.01	3.81±1.36	4.57±1.57^b^	4.44±1.40^b^	4.31±1.49^b^	4.44±1.44^b^	0.0045
								
24–48 h	Frequency	0.82±0.53	0.77±0.60	1.03±0.66	0.71±0.58	0.68±0.64	0.80±0.63	0.1373
	Consistency	3.46±0.87	3.63±1.38	3.93±1.35	3.64±1.14	3.481.19	3.42±1.36	0.5420

Abbreviations: ITT, intention to treat; scFOS, short-chain fructo-oligosaccharides.

Data is expressed in mean±s.d.

a Number of bowel movements per 24 h.

b Significant increase (Dunnett's test) in gastrointestinal symptoms scores compared with 35 g dextrose, *P*<0.05. Other observed differences (Tukey's test) for stool frequency at 24 h: maltitol 35 vs dextrose 24–scFOS 11 (*P*=0.0154); maltitol 30–scFOS 5 vs dextrose 24–scFOS 11 (*P*=0.0069); for stool consistency at 24 h maltitol 35 (*P*=0.0023), maltitol 30–scFOS 5 (*P*=0.0137), maltitol 24–scFOS 11 (*P*=0.0374) and maltitol 17.5–scFOS 17.5 (*P*=0.0085) vs dextrose 24–scFOS 11.

c Consistency score from Bristol scale (1: separate hard lumps to 7: watery, entirely liquid).

**Table 4:** AUC_(0–120min)_ and Cmax of plasma glucose and insulin for 2 h after consumption of the dairy desserts containing 35 g of dextrose or different mixtures of maltitol and scFOS in the ITT population (*n*=18)

	*Control*	*Dextrose 24 scFOS 11*	*Maltitol 35*	*Maltitol 30 scFOS 5*	*Maltitol 24 scFOS 11*	*Maltitol 17.5 scFOS 17.5*	*Dessert effect (*P-*value)*
*Glucose*							
AUC (mmol min/l)	110.4±56.4	70.1±48.3	60.7±51.9^a^	51.6±34.1^a^	43.6±46.0^a^	31.0±26.3^a^	<0.0001
Cmax (mmol/l)	8.1±1.6	7.3±1.3^a^	6.8±1.1^a^	6.5±0.9^a^	6.0±0.8^a^	5.9±0.8^a^	<0.0001
							
*Insulin*							
AUC (mU min/l)	4224.1±1616.9	3221.3±1566.7^a^	2657.8±1390.8^a^	2285.4±765.6^a^	1752.9±990.2^a^	1681.9±825.9^a^	<0.0001
Cmax (mU/l)	94.9±36.0	85.6±32.8	65.3±31.0^a^	53.6±18.8^a^	40.4±17.7^a^	43.1±18.1^a^	<0.0001

Abbreviations: AUC, area under curve; ITT, intention to treat; scFOS, short-chain fructo-oligosaccharides.

Data are expressed in mean±s.d.

a Significant decrease in glucose or insulin AUC or Cmax compared with control (35 g dextrose, *P*<0.05) (Dunnett s' test).

## Notes

FR, CH and AW are employees of Tereos Syral. The remaining authors declare no conflict of interest.
