# Supplementary material for: Digestive tolerance and postprandial glycaemic and insulinaemic responses after consumption of dairy desserts containing maltitol and fructo-oligosaccharides in adults
Source: Eur J Clin Nutr. 2014 Mar 19;68(5):575–80. doi: 10.1038/ejcn.2014.30 (PMC4013556; doi:10.1038/ejcn.2014.30)
Supplement: Supplementary Table 1 [file ejcn201430x1.doc]

Supplemental Table 1: Total and individual scores of gastrointestinal symptoms in the 24h and 48h following consumption of dairy desserts containing 35g dextrose (control dessert) or different mixtures of maltitol and scFOS in the PP population (n=32). Each symptom is scored between 1 and 10; data is expressed in arbitrary unit mean ±SD.

|  |  | Control | Dextrose 24  scFOS 11 | Maltitol 35 | Maltitol 30  scFOS 5 | Maltitol 24  scFOS 11 | Maltitol 17.5  scFOS 17.5 | Dessert effect (P value) |
| --- | --- | --- | --- | --- | --- | --- | --- | --- |
| 0-24h | Total score | 3.09±4.31^a^ | 6.38±5.70 ^b^ | 8.69±5.26 ^bc^ | 8.41±7.05 ^bc^ | 9.72±7.00 ^bc^ | 10.38±7.47 ^c^ | <0.0001 |
|  | Flatulence | 1.19±1.51 | 2.25±2.30 | 3.44±2.40* | 2.72±2.47* | 3.69±2.38* | 3.94±2.27* | <0.0001 |
|  | Borborygmi | 0.69±1.33 | 1.59±2.06 | 2.03±2.12* | 2.44±2.45* | 1.91±2.35* | 2.34±2.34* | <0.0001 |
|  | Bloating | 0.66±1.70 | 1.31±2.04 | 1.53±2.09 | 1.78±2.20 | 1.94±2.44* | 2.17±2.72* | 0.0341 |
|  | Discomfort | 0.56±1.70 | 1.22±2.20 | 1.69±2.42 | 1.47±2.96 | 2.19±3.00* | 1.97±2.60* | 0.0209 |
| 24-48h | Total score | 0.94±1.75 | 2.38±3.68 | 3.19±4.64 | 2.38±3.13 | 2.31±3.45 | 2.53±4.17 | 0.0831 |
|  | Flatulence | 0.29±0.82 | 0.75±1.46 | 1.199±1.77* | 1.09±1.61* | 1.06±1.56* | 1.09±1.75 | 0.0444 |
|  | Borborygmi | 0.32±0.83 | 0.34±0.97 | 0.66±1.33 | 0.72±1.42 | 0.47±1.16 | 0.53±1.27 | 0.4659 |
|  | Bloating | 0.13±0.56 | 0.53±1.22 | 0.59±1.52 | 0.28±0.81 | 0.50±1.05 | 0.56±1.39 | 0.4284 |
|  | Discomfort | 0.19±0.60 | 0.75±1.61 | 0.75±1.61 | 0.28±0.92 | 0.28±0.81 | 0.34±1.21 | 0.1049 |

^a^ Desserts not sharing the same letter are significantly different (p<0.05, Dunnett’s post hoc test vs the Control or Tukey’s test among the 5 other mixtures).

* Significant increase (Dunnett’s test) in gastrointestinal symptom scores compared to 35g dextrose, p<0.05.
