# Supplementary material for: Digestive tolerance and postprandial glycaemic and insulinaemic responses after consumption of dairy desserts containing maltitol and fructo-oligosaccharides in adults
Source: Eur J Clin Nutr. 2014 Mar 19;68(5):575–80. doi: 10.1038/ejcn.2014.30 (PMC4013556; doi:10.1038/ejcn.2014.30)
Supplement: Supplementary Table 2 [file ejcn201430x2.doc]

Supplemental Table 2: Stool frequency and consistency evaluated by the Bristol stool scale in the 24h and 48h following consumption of dessert creams containing 35g of dextrose or different mixes of maltitol and scFOS in the PP population (n=32). Data are expressed in mean±sd

|  |  | Control | Dextrose 24  scFOS 11 | Maltitol 35 | Maltitol 30  scFOS 5 | Maltitol 24  scFOS 11 | Maltitol 17.5  scFOS 17.5 | Dessert effect (P value) |
| --- | --- | --- | --- | --- | --- | --- | --- | --- |
| 0-24h | Frequency^1^ | 1.25±0.84 | 1.44±0.84 | 1.66±0.79* | 1.72±0.89* | 1.88±0.94* | 1.81±0.93* | 0.0016 |
|  | Consistency | 3.72±1.03 | 3.94±1.30 | 4.34±1.43 | 4.23±1.45 | 4.39±1.40 | 4.51±1.55 | 0.1084 |
| 24-48h | Frequency | 0.81±0.54 | 0.75±0.57 | 0.78±0.61 | 0.63±0.61 | 0.66±0.55 | 1.00±0.67 | 0.0947 |
|  | Consistency | 3.46±0.89 | 3.55±1.41 | 3.45±1.41 | 3.56±1.20 | 3.55±1.15 | 3.88±1.35 | 0.9530 |

^1^ Number of bowel movements per 24h

* Significant increase (Dunnett’s test) in gastrointestinal symptoms scores compared to 35g dextrose, p<0.05.
